# Browsing repeats in genomes: Pygram and an application to non-coding region analysis

**DOI:** 10.1186/1471-2105-7-477

**Published:** 2006-10-26

**Authors:** Patrick Durand, Frédéric Mahé, Anne-Sophie Valin, Jacques Nicolas

**Affiliations:** 1 IRISA/INRIA, Campus de Beaulieu, 35042 Rennes Cedex, France; 2 ECOBIO, CNRS UMR 6553, Campus de Beaulieu, 35042 Rennes Cedex, France; 3 Institut Curie, Dept transfert, Quadrilatère historique Hôpital Saint Louis, Porte 13, 1 rue Claude Vellefaux, 75010 Paris, France

## Abstract

**Background:**

A large number of studies on genome sequences have revealed the major role played by repeated sequences in the structure, function, dynamics and evolution of genomes. In-depth repeat analysis requires specialized methods, including visualization techniques, to achieve optimum exploratory power.

**Results:**

This article presents Pygram, a new visualization application for investigating the organization of repeated sequences in complete genome sequences. The application projects data from a repeat index file on the analysed sequences, and by combining this principle with a query system, is capable of locating repeated sequences with specific properties. In short, Pygram provides an efficient, graphical browser for studying repeats. Implementation of the complete configuration is illustrated in an analysis of CRISPR structures in *Archaea *genomes and the detection of horizontal transfer between *Archaea *and *Viruses*.

**Conclusion:**

By proposing a new visualization environment to analyse repeated sequences, this application aims to increase the efficiency of laboratories involved in investigating repeat organization in single genomes or across several genomes.

## Background

Some years ago, genomes were considered as static objects containing an informative part, the coding sequences, representing only a small percentage of the total genome, and a part referred to as "junk DNA" that was generally free of any annotation. It is now widely acknowledged that genomes must be considered from a more dynamic point of view, involving the study of the many " copy" events that occur during evolution, while covering not only coding genes, but non-coding sequences as well. A large number of *in silico *studies have revealed that repetitive sequences play an important role in the structure, function, dynamics and evolution of genomes in *Archaea *[1,2], *Bacteria *[3,4] and *Eukarya *[5-7]. It is well known, for instance, that proteins are combinations in a finite set of domains that represent basic structural units whose arrangements determine a wide variety of functions. Other classes of repeats, such as transposable elements, allow mobile elements to move around a genome, and have a major impact on the evolution of sequences [8]. DNA palindromes, a particular form of repeat, are widespread in human cancers. Other repeats in centromeric or telomeric regions of chromosomes seem to endow a certain robustness to the sequences during replication. Repeats may be strictly conserved through evolution, as revealed by comparisons of human, mouse, rat, chicken and dog genomes [9,10]. Complex mechanisms such as chromosome segment duplications, or even whole genome duplications, are thought to occur, explaining genome evolution [11,12]. Converging studies of human and other genomes have also revealed that variations in the number of occurrences of particular repeats may be an important factor responsible for diseases such as diabetes, epilepsy, fragile-X mental retardation and myotonic dystrophy diabetes [13,14]. From a technical point of view, repeats are the source of many difficulties encountered in assembling or comparing sequences, requiring their extraction from these sequences. For these and other reasons, the analysis of repetitive sequences is an essential step in genome assembly, annotation and analysis.

At the core of life information, there exists an outstanding opportunity to analyse the genomic structure by deciphering its content in repeated sequences. The exhaustive analysis of 360 published complete sequences from *Archaea, Bacteria *and *Eukarya *genomes (data from Genome OnLine Database [15]) has revealed that most of them, especially in *Eukarya*, have a genomic content consisting of large proportions of repeats. Revealing the structure of sequences as an assembly of elementary repeated sequences is thus a task of utmost importance.

An important goal in computational molecular biology is therefore finding repeats of biological interest, i.e. repeats that have a role in genome structure and function. Practical libraries of repeats have been established in an attempt to collect prototypical sequences and group them into families, either for a large set of genomes or for a particular species [16-19]. To achieve this goal using computational methods, the problem consists in giving a precise definition of a "repeat". In the biological literature, three main classes of repeats are proposed: *tandem repeats *(consecutive copies of patterns), *duplicated segments *(which include genes and chromosome segment duplications) and *interspersed repeats *(which include transposons). Tandem repeats are thought to have originated by slippage of a replicated chromosome against its template. The patterns in tandem repeats are *k*-mers, *k *being generally less than 5 (micro-satellites), but sometimes far greater (up to several thousand base pairs long, with a total size that can represent several uninterrupted megabases). The number of repeats for a given satellite may differ between individuals. Therefore, they can be used for DNA fingerprinting or to provide information about paternity.

Microsatellites, also known as short tandem repeats (STR), have a repeat unit that is 2 to 10 bp long, with the entire repetitive region spanning less than 200 bp. Minisatellites are generally GC-rich repeats that range in length from 10 to over 100 bp with total length ranging from 1 kb to 20 kb. Duplicated segments are large intra- or interchromosomally DNA segments, ranging from 41 to 655 kb in size and likely to result from replication accidents. These events result in the duplication of gene clusters. Interspersed repeats or mobile elements are DNA sequences located in dispersed regions in a genome, produced by mechanisms such as DNA recombination. The gene pool of a species consists of DNA sequences in a network linked by gene conversion events. This type of repetitive sequence plays the role of uncoupling the network, thereby allowing new genes to evolve. In mammals, the most common mobile elements are LINEs for interchromosomal uncoupling (length ≃ 6–7 kb) and SINE for intrachromosomal uncoupling (total length ≃ 300 bases). The first mobile elements were discovered by Barbara McClintock in the 1940s in studies on corn. Subsequently, they were found in all kinds of organisms. Classifications such as these provide a better understanding of the biological processes at hand during genome evolution. But since they are based on current limited biological knowledge, these definitions introduce some bias in the type of repeats targeted by the analysis, and also introduce complexity in the algorithms used to locate them, especially when considering error-prone repeats. PILER, [20] represents the current state of the art in this respect, where four classes of biological repeats are defined. Classes TA (tandem array) and DF (dispersed family) correspond to the previously cited tandem repeats and interspersed repeats, respectively. The other two classes are pseudosatellites (PS), which are clustered elements in the genome that are not tandem repeats, and terminal repeats (TR), which are copies of the same element located at the termini of a duplicated element.

A number of formal definitions have been proposed to capture the essence of observable repeats. A vast amount of literature covers this problem, and essentially three categories of formal repeats have been proposed: words, contiguous repeats and structured repeats. The first category tries to distinguish among repeated words those that include all other ones and are thus representative of the whole set of repeats. It mainly uses a maximization criterion, such as the longest repeats [21,22] and maximal repeats [23,24]. The second category introduces a basic model to achieve a closer approximation of observed repeats, since natural repeats in genomic sequences usually present many variations of close basic repeat units. Certain authors propose to look for trains of contiguous repeats such as tandem arrays (e.g., [25-27]), or pairs of repeats at a fixed distance (e.g, longest repeats with a block of don't cares [28], maximal pairs with bounded gap [29]), or to introduce an edit distance or a similarity score to take into account local variations (e.g k-mismatch repeats [30] and approximate tandem repeats [31]). Finally, the third category contains sophisticated repeat models that include all the previous notions and are designed to discover the complex word arrangements that occur with a minimum frequency. A structured motif consists of an ordered collection of *p *> 1 parts separated from one another by spacers, the length and distance between parts being bounded with given Min and Max values [32-34]. This kind of repeat seems of particular interest in studying non-coding sequences in gene expression and regulation.

Among these formal definitions, the notion of exact maximal repeat is quite attractive, since it is at the core of all others. It only focuses on sequences present in the two largest common blocks, with no possible extension to the right or left, and with no biological *a priori*. Maximal repeats have nice properties: they can be computed in linear time using a suffix-tree-based algorithm, their number is linear (at most *n *kinds of exact maximal repeats in a sequence of size *n*), and they can be used as basic blocks to compute error-prone repeats [30].

Associated visualization techniques play a fundamental role in analysing these numerous repeats, and various kinds of tools displaying repeats at genome level have been proposed in the past few years. Among them are dotplots [35], landscapes [36], chaos games [37], percent identity plots [38], repeat graphs [30] and BARD [39]. Interpreting the views created using these tools is quite difficult, however, especially for large genomes, since most of them rely on displaying repeat pairs. They do not usually provide convenient zooming features to analyse regions of particular interest. Tools like dotplot, chaos game and BARD still can only be used on pairwise genome sequence alignments, and, because they only work at sequence level, become difficult to use as the sequence size and/or number of repeats increases. Moreover, they are not capable of summarizing the hierarchical organization of repetitive structures in a convenient way so that they can be interpreted by the end users.

This paper introduces the pyramid diagram, or *pygram*, designed to provide an abstract representation of the organization of repeated structures in genomic sequences. The theoretical foundation of *pygrams *is similar to sequence landscapes, which display all exact maximal repeats in a picture. But the *pygram *improves the original sequence landscape visualization in several ways. Aside from various practical improvements (two-strand display, zoom lenses), *pygram *offers several new features, including frequency visualization and multigenome repeat analysis. Most important, *pygram *visualization is closely associated with a query system designed to locate repeats that share specific properties. When combined, the query system and visual interface provide an efficient repeat browser that is useful for discovering unexpected structures in genomes.

## Results and Discussion

### Pyramid Diagram (Pygram) description

A pygram for a genome sequence *S *of length *n *is a bi-dimensional plot where *S *and all its exact maximal repeats (**eMR**) are mapped along the *x*-axis. Given an *x*-axis magnifying factor *k *and a *y*-axis magnifying factor *l*, mapping is defined as follows: the *i*^*th *^nucleotide of *S *is located at position (*i/k*,0), and the eMR of size *m *located at position *i *within *S *corresponds to the interval [*i/k*,(*i*+*m*)/*k*] on the *x*-axis. The size *m *eMR located at position *a *within *S *is symbolized in the diagram by an isosceles triangle (**a pyramid**) of height *δ m/l*. *δ *is either '+1' for an eMR located on the normal (N) strand of *S*, or '-1' for an eMR located on the reverse complement (RC) strand of *S*.

Since focus will be placed on eMRs in the rest of this paper, and most of the presentation does not depend on the kind of repeat used, the simpler term "repeat" will be used instead of "exact maximal repeat". It is first important to emphasize three basic facts about managing two DNA strands to avoid confusion in the interpretation of results.

• A single coordinate system is used for both strands, i.e. all repeat coordinates, whether they are located on N or RC strands, are computed relative to the N strand.

• The word on the reverse complement strand must be read as usual in the reverse direction. On the *pygram *each pyramid has an associated colour computed from the corresponding eMR sequence, ensuring that each repeat has its own specific colour. Consequently, all occurrences of the same repeat will have the same colour on both strands.

• The definition of an eMR is symmetrical with regards to N and RC strands: if a word w is an eMR, then word w¯c
 MathType@MTEF@5@5@+=feaafiart1ev1aaatCvAUfKttLearuWrP9MDH5MBPbIqV92AaeXatLxBI9gBaebbnrfifHhDYfgasaacH8akY=wiFfYdH8Gipec8Eeeu0xXdbba9frFj0=OqFfea0dXdd9vqai=hGuQ8kuc9pgc9s8qqaq=dirpe0xb9q8qiLsFr0=vr0=vr0dc8meaabaqaciaacaGaaeqabaqabeGadaaakeaadaqdaaqaaiabdEha3baadaahaaWcbeqaaiabdogaJbaaaaa@2FB0@, the reverse complement of w, is also an eMR. The display of an eMR along one strand is always mirrored with an eMR of the same size on the other strand.

**Figure 1:**
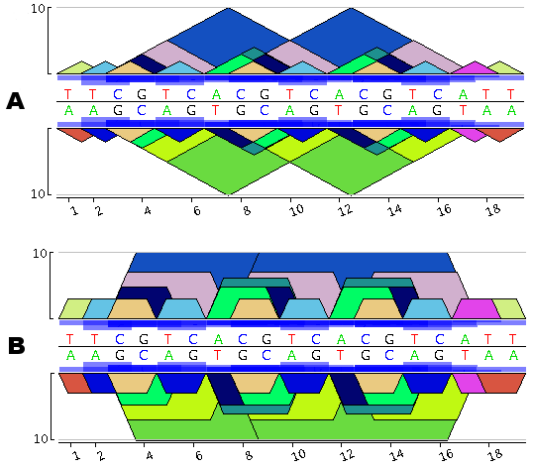
**Construction of a *Pygram***. A. *Linear pygram *of the DNA sequence TTCGTCACGTCACGTCATT. The direct strand representation is above the horizontal black line, while the reverse complementary strand is below this line. The *x*-axis corresponds to the N strand coordinate system and the *y*-axis to eMR size. Blue boxes located in front of the sequence indicate the eMR frequency. B. *Logarithmic Pygram *of the same DNA sequence where the *y*-axis scale is logarithmic.

Figure 1 presents the *pygram *for the short DNA sequence 5'-TTCGTCACGTCACGTCATT-3'. According to Gusfield's eMR, definition, the sequence TTCGTCACGTCACGTCATT contains 13 different eMRs, namely: A, C, G, T, AT, CG, TC, TT, ACG, CGT, ACGT, CGTCA, CGTCACGTCA. Note that some eMRs may overlap or even include other eMRs, revealing the structural organization of these repeats. To ensure that all repeats in this sequence are visible, those that are included in other ones are displayed on top. As mentioned in the third remark above, the entire diagram is symmetrical. For instance, eMR, 5'-CGTCACGTCA-3' (in Figure 1A this eMR, starts at positions 3 and 8) is represented by a blue triangle that is always above a green triangle corresponding to the complementary eMR, 3'-TGACGTGACG-5' (this eMR, ends at positions 3 and 8). In Figure 1A, CGT is a repeat represented by a dark blue pyramid with three occurrences on the direct strand 5'-CGT-3' at positions 3, 8 and 13, and two occurrences on the reverse strand 3'-CGT-5' at positions 9 and 14. Therefore, if a word is a biological palindrom, it immediately appears in the diagram as a pyramid mirrored in the other strand using a pyramid of the same colour. For instance, CG is displayed using a mirrored orange triangle, since 5'-CG-3' matches 3'-CG-5' and ACGT appears at positions 7 and 12, represented by the same dark green colour on both sides. Finally, the repeat frequency is represented using boxes proportional in size to the frequency in question, these boxes being displayed in the middle of the *pygram *at each occurrence of the repeat. *Pygrams *can be drawn using either a linear or a log10 *y*-axis coordinate system. The latter is more apt at revealing the structure of small repeats contained in larger ones. But if a logarithmic *y*-axis were to be used while keeping the triangles displayed, small repeats would no longer be completely included within any repeats containing them, and the display of eMR hierarchical structures would be lost. To restore this visualization feature, *log-pygrams *no longer display triangles, but show trapezoids instead (Figure 1B).

Since the basic idea behind the *pygram *is to display all exact maximal repeats, *pygrams *may be considered as a rational reconstruction of landscapes [36], fully characterizing the structure that is displayed without requiring the computation of intermediate repeats. Landscapes do indeed display maximal repeats, where the scope of the right triangles is such that increasing the corresponding subword to the left or right removes at least one occurrence of the repeat in the extended subword. This provides a precise definition of maximal repeats.

### Producing pygrams and browsing repeats

The first step in creating a *pygram *consists in producing the complete set of repeats. Since the repeat structures are to be analysed either within a single sequence or across several sequences, Gusfield's eMR detection algorithm [23] was implemented on a generalized suffix tree (see *Methods*).

The second step in constructing a *pygram *consists in creating an indexed representation of the complete set of eMR occurrences. Indexing aims to order repeats along the sequences, so that *pygrams *can be created efficiently. Indexing also improves browsing speed when checking specific repeat properties, such as frequency, size, location (normal vs. reverse complement strand), and conservation between two or more sequences. This close association between repeat visualization and querying provides an efficient browsing function for in-depth analysis of repeat organization at various levels, from the highest level (i.e. the complete sequence) to the lowest level (a single nucleotide).

The following discussion illustrates the browser capabilities through two case studies. The first shows how to detect and analyse *Clustered Regularly Interspaced Short Palindromic Repeats *(CRISPR; [40]). The second presents an analysis of the horizontal transfer of DNA sequences between two genomes.

### Visual analysis of repeat organization

**Figure 2:**
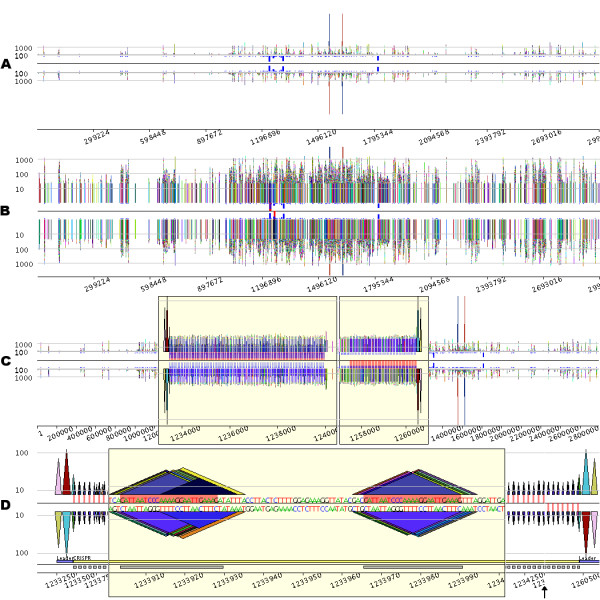
**Visual investigation of CRISPRs in the *Sulfolobus solfataricus *P2 genome (RefSeq entry NC_002754)**. From A to D, please note that the *x*-axis scale varies, depending on the degree of magnification. A. *Linear pygram *of the complete genome sequence. The normal (N) strand view is above the black horizontal line, and the reverse complement (RC) strand view is below this line. The *x*-axis corresponds to the sequence coordinate system. The *y*-axis corresponds to eMR size, and horizontal grey lines representing eMRs containing 100 and 1000 nucleotides are displayed on both N and RC views. The small blue boxes located between the black line and the N and RC views indicate eMR frequency. B. *Logarithmic Pygram *of the same genome. The small red boxes located between the black line and the two-strand views highlight the most frequent repeat in the genome. C. *Pygram *with two zoom lenses. The first yellow box represents a lens that magnifies the *x*-axis of the sequence region 300 times, between coordinates 1,233,000 and 1,240,500. The second lens magnifies the *x*-axis of the sequence region 500 times, between coordinates 1,257,000 and 1,261,500. Since the *y*-axis scale is linear, the *y*-axis was magnified 20 times in the lens regions for a better view of small repeat organization (note the shift of the *y*-axis grey lines; inside the lens, the two remaining grey lines are for repeats containing 10 and 100 nucleotides). The small red boxes located between the black line and the two-strand views highlight the most frequent repeat in the genome. D. Details of two CRISPRs. The *pygram *displays the first 1500 nucleotides of the first CRISPR presented in Figure C, followed by the last 700 nucleotides of the second CRISPR from Figure C; for the sake of clarity, the black vertical arrow has been added to mark the 26.5 kb gap separating the two CRISPRs. The grey horizontal line inside the lens marks repeats containing 10 nucleotides, whereas the grey lines outside the lens designate repeats containing 10 and 100 nucleotides. In the centre of the *pygram*, the red rectangle highlights the most repetitive eMR identified in the genome. The main constituents of CRISPR are annotated just above the *x*-axis numbers: the leader sequence and CRISPR units are underlined with blue and grey rectangles, respectively.

Figure 2 displays a *pygram *representing 92,424 occurrences of 16,118 different repeats containing at least 20 nucleotides located on both strands of the 2.83 Mb genome sequence of *Sulfolobus solfataricus *P2 (RefSeq entry NC_002754). Due to the high density of information, repeats do not appear as pyramids, but as vertical bars (Figure 2A). Several large repeats featuring more than 1000 nucleotides can be detected. Among them, the largest one (6521 nucleotides) clearly appears on the *pygram *as the highest bar, one occurrence being on the N strand, the other being on the RC strand. This genome also contains a large number of repeated sequences, but this feature does not appear very well in Figure 2A. The log-*pygram *of the genome (Figure 2B) more accurately reveals the overall organization of the repeated sequences, dispersed along the entire genome. Among these numerous repeats, *S. solfataricus *contains five short regions with high repeat frequency, as highlighted by the frequency bars. Since these bars are displayed at each position on the eMR, their nearness demonstrates that the corresponding eMR occurrences are located close together.

To further investigate these frequently repeated regions, a *pygram *with two zoom lenses is presented in Figure 2C (the *Methods *section gives a detailed description of the implementation and graphical features of the *pygram *viewer). The lenses magnify two frequently repeated regions previously identified on the complete genome *pygram*, where numerous repeats appear to be organized in arrays of spaced tandems. Zooming in further (Figure 2D) reveals that the two regions share a similar structure. The tandem repeat in the first lens is preceded by two large repeats, symbolized by pink and red triangles on the N strand. Identical large repeats can be observed on the opposite strand, downstream of the tandem repeat displayed in the second lens. Instances of a 25-nucleotide-long repeat appear regularly spaced, consecutive occurrences being separated by non-repetitive sequences. This type of structure has already been observed in this genome [41] and is known as CRISPR.

CRISPRs are a very peculiar family of repeated sequences found in *Archaea *and *Bacteria *genomes [40]. Their remarkably constant structure consists of short sequences from 21 to 37 nucleotides long, repeated almost exactly, and referred to as 'units', separated by similarly sized non-repetitive sequences, called 'spacers' (Figure 2D). In most species with two or more CRISPR loci, these loci are flanked on one side by a common leader sequence of 300–500 nucleotides. In Figure 2D, the leader sequence is delineated by the previously mentioned pink and red large repeat.

Each CRISPR unit appears as a group of co-occurring repeats that differ by only a few nucleotides. This is the result observed when a sequence, here the CRISPR unit, is repeated several times with point mutations. If a maximal repeat occurrence mutates at some point, it results in two included maximal repeats overlapping at the point of mutation: if *aubvc *is an eMR, *a*, *b *and *c *being letters and *u *and *v *two words, then a mutation from *b *to *d *leads to eMRs *aud *and *dvc*. Visualizing all eMRs detected on both strands of a complete genome can therefore be used to identify error-prone repeated sequences.

### Querying the eMR index to locate exceptional repeats

Another way to target the presence of specific repeated structures consists in querying the eMR index file, then interpreting query results on a *pygram*. Such queries can be simple, consisting of searching for the most frequent repeats, or complex, as in the case of searching for specific repeat patterns. In the case of *S. solfataricus*, querying the index to answer the first question returns a 25-nucleotide eMR repeated 151 times in the complete genome, 103 occurrences being located on the N strand and 48 on the RC strand. This information can be drawn directly from *pygrams *(Figures 2B to 2D), where the specific eMR is highlighted in red on the centre frequency line. These *pygrams *immediately show that the most repeated eMR is located exclusively within two different CRISPRs, forming the most conserved element of the repeated units (Figure 2D).

**Figure 3:**
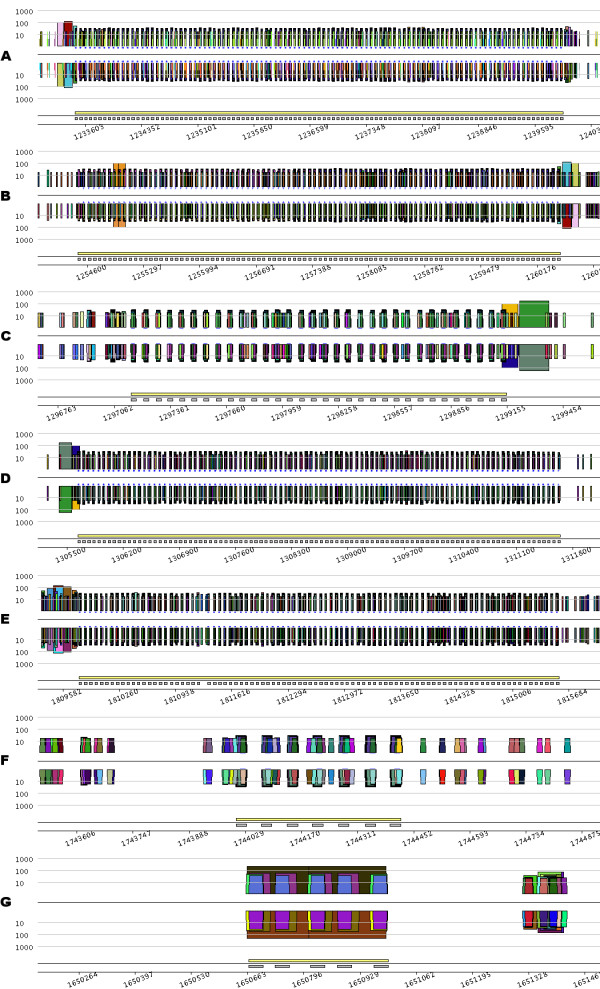
***Pygrams *resulting from running a query on the eMR index file using a CRISPR model**. From A to G, the different parts of the figure are the *pygrams *displaying the seven regions detected by querying the eMR index file using a CRISPR model. Note that the *x*-axis scale is different for each part of the figure, depending on CRISPR size. Each picture is a *log-pygram *displaying all repeats containing 20 nucleotides or more, identified on both strands of the genome. Each *pygram *displays the location of the entire CRISPR (yellow rectangle) and its repeated units (grey rectangles) just above the *x*-axis.

Searching for more CRISPRs in the *S. solfataricus *genome can be performed by querying the eMR, index file using a CRISPR model defined on the basis of eMR properties. More precisely, the index file can be queryied to select repeats based on their size, number of occurrences and location in the sequence. In Figure 3, the index file was queried to locate repeats ranging from 20 to 40 nucleotides, repeated at least 4 times, with consecutive repeat occurrences being separated by no more than 70 nucleotides. The eMR occurrences reported by the query are distributed among seven different regions in the genome. Visualization of the corresponding *pygrams *(Figure 3) led to the identification of the six well-known CRISPRs published previously [41]; *pygrams *from Figures 3A and 3B display the CRISPRs presented in the previous section (see Figure 2).

The *pygrams *presented in Figure 3 show that some CRISPRs have a similar leader sequence. The left side of Figure 3A has two large repeats (pink and red) that correspond to the right side of Figure 3B, but on the opposite strand. Likewise, repeats at the end of Figure 3C (orange and green) match those at the beginning of Figure 3D. Considering the visual properties of these pictures, it is worth noting that *pygrams *using a logarithmic *y*-axis (Figure 3) render the CRISPR, structure better than *linear-pygrams *magnified using the zoom lens (Figure 2).

Among the various CRISPRs displayed in Figure 3, two are questionable (Figures 3F and 3G), since they do not fit the repetitive structure observed in Figures 3A to 3E. The CRISPR in Figure 3F is quite short, with its seven repeated units, and there is no detectable leader sequence. However, querying the index file to retrieve all eMR occurrences forming the units in this CRISPR reveals that these repeats are also located within the CRISPR units from Figure 3E, and nowhere else on the genome. Therefore, the CRISPR in Figure 3F should be a real one, even if it is quite short. The relationship between CRISPRs from Figures 3E and 3F remains unclear.

The structure presented in Figure 3G is an example of what could be a false positive reported when querying the index file using the above-mentioned CRISPR model. Even if some repeats are organized like CRISPR units, the overall structure is repeated, as revealed by the large brown trapezoid on the N strand, and the eMR forming that structure cannot be found anywhere else in the genome. This example illustrates the advantages of using *pygrams *to visually interpret the results of a computational method that predicts the presence of specific patterns of repeated structures.

**Figure 4:**
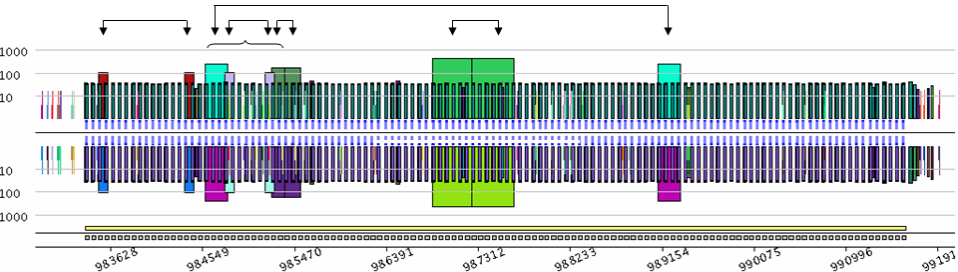
***Pygram *of the 8 kb CRISPR from *Methanothermobacter thermautotrophicus *str. Delta H (RefSeq entry NC_000916)**. The picture shows a *log-pygram *representing all repeats with 20 nucleotides or more, identified on both strands of the genome. Arrows point out the corresponding parts of the 5 internal duplications, while the horizontal curly parenthesis highlights the overlap of three out of five internal duplications. The bottom line of the *pygram *displays the location of the full CRISPR (yellow rectangle) and its repeated units (grey rectangles).

The conventional CRISPR model is based on repeated units separated by non-repeated sequences, known as spacers [40]. Figure 3B, however, shows a short duplication on the left side of the structure, depicted by the orange trapezoid containing roughly 100 nucleotides. Duplication involves two units and one spacer that are exact repeats. Other exceptional internal duplications in CRISPRs can be observed in *Archaea *genomes. For instance, the large 8 kb CRISPR from *Methanothermobacter thermautotrophicus *str. Delta H (RefSeq entry NC_000916) contains five duplications (Figure 4). Two of them look like tandem duplications, but closer examination shows that they overlap by one CRISPR unit. The other three duplications show partial overlaps, as pointed out in Figure 4. These examples illustrate the ability of a *pygram *to reveal the complex hierarchical organization of repeated sequences where large repeats contain smaller but highly organized repeated sequences. Another example of these hierarchical structures is shown in Figure 3G.

### Analysing repeats across two genome sequences

It was recently reported that *S. solfataricus *CRISPRs contained foreign genetic elements from the *SIRV1 *virus [2]. The authors have suggested that these particular CRISPRs, which contain *SIRV1 *foreign DNA, could be involved in the known immunity of *S. solfataricus *against *SIRV *viruses.

**Figure 5:**
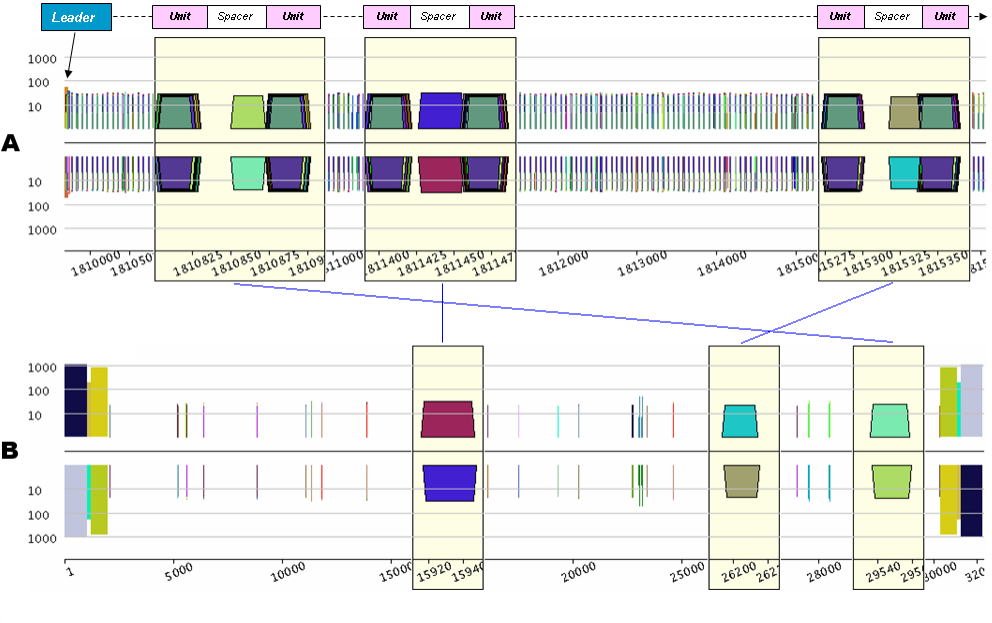
**Detection of SIRV1 viral DNA in *S. solfataricus *CRISPR spacers**. A. *Log-pygram *of *S. solfataricus *CRISPR ranging from coordinates 1,809,680 to 1,815,570. Each lens shows a single spacer surrounded by the peculiar *S. solfataricus *conserved repeats forming the CRISPR units. For the sake of clarity, the main CRISPR constituents (leader, unit and spacer) and orientation (thick black arrow) have been sketched above the *pygram*. B. *Log-pygram *of the *SIRV1 *genome sequence. Each lens displays the repeats, containing from 21 to 31 nucleotides, located in the CRISPR spacers of *S. solfataricus*.

This type of DNA transfer can be detected using the *pygram *application. It consists in looking for repeats for which one copy is located on the archaeal genome, the other copy being located on the virus genome. Using this method, five *SIRV1 *sub-sequences were detected within the *S. solfataricus *genome (on the complementary strand). Figure 5 shows three of them, and it is interesting to note that these sequences are located within the spacers of a CRISPR on the archaeal genome sequence. None of the three *SIRV1 *short sequences were detected anywhere else in the archaeal genome.

These observations are consistent with data reported by Mojica *et al*. [2], although the present study failed to locate one out of the six known *SIRV1 *sub-sequences integrated in *S. solfataricus *as reported by these authors. This can be explained by the fact that the study only covered recognition of repeats containing 20 nucleotides or more, whereas Mojica *et al*. used BLAST, which is capable of recognizing shorter sequences. The *pygram *method may still be used to locate this particular sub-sequence, however, by lowering the repeat recognition size to 10 nucleotides.

### Comparison with existing visualization methods

We generated a *dotplot, percent identity plot *(PIP), *repeat graph *and *pygram *from the same sequence to compare these visualization techniques in studying repeated sub-sequence organization within genome sequences. The sequence analysed here is the 2.83 Mb genome of *S. solfataricus*.

**Figure 6:**
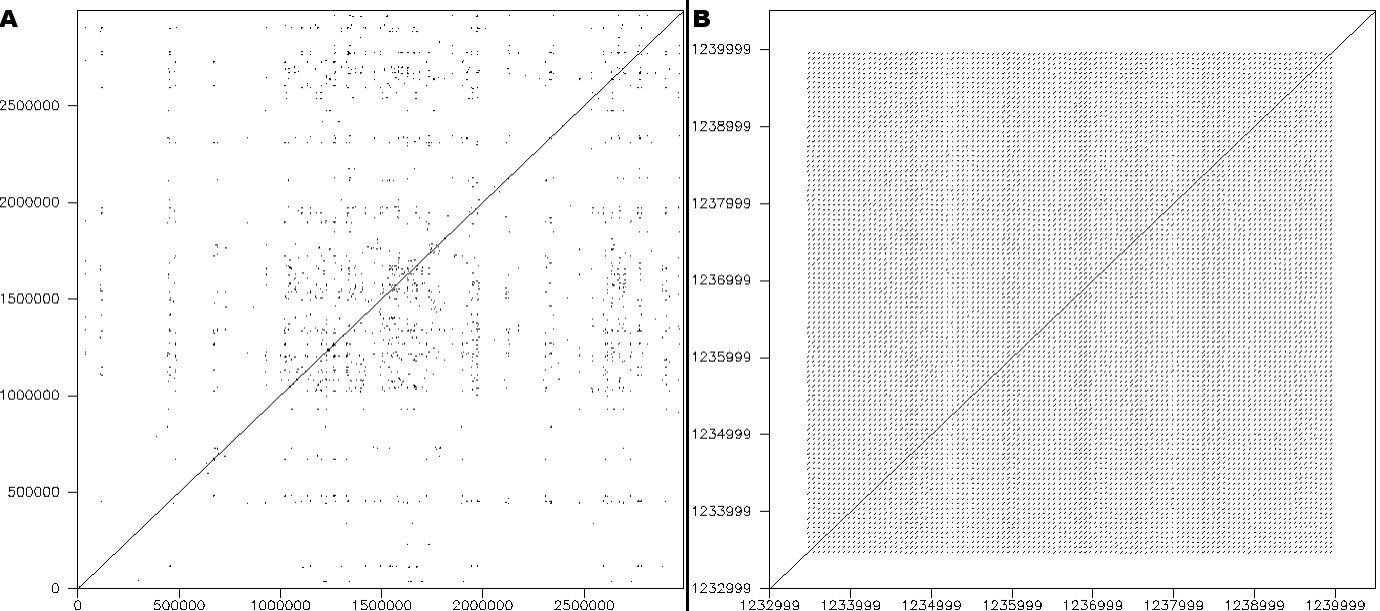
**Dotplot**. A. Dotplot of the 2.83 Mb sequence from *S. solfataricus*. B. Dotplot of the *S. solfataricus *sequence region from 1,233,000 to 1,240,000, containing a CRISPR.

The dotplot was produced using *dottup *from the *EMBOSS *package [42]. It displays the location of all exact hits of sub-sequences measuring 20 bp or more (Figure 6a). The dotplot provides a general overview of the genome sequence compared with itself, where it appears that a large number of repeated sub-sequences exist. The interpretation of a dotplot, however, is limited to the general location of repeated segments, and two figures are actually required: one to compare the N strand with itself and another to compare the N strand against the RC strand. The result does not provide any information regarding the size and organization of these repeats, even when zooming in on regions of interest such as CRISPRs (Figure 6b). *Pygrams *reveal this kind of information more precisely, from a general view down to more detailed images (Figure 2). In the general view, repeat frequencies underline regions of particular interest, and the magnified views of these regions clearly depict how they are organized.

**Figure 7:**
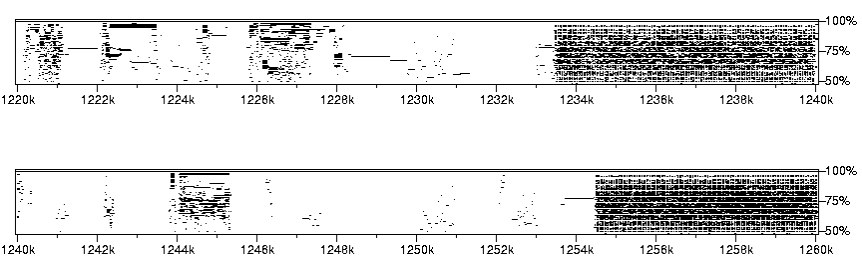
**Percent Identity Plot (PIP)**. Percent Identity Plot of the 2.83 Mb sequence from *S. solfataricus*. Only the sub-sequence ranging from coordinates 1.22 Mb to 1.28 Mb is shown, since the complete PIP spans 30 pages.

A PIP is useful in determining the degree of conservation between two or more sequences. The PIP is a 2D diagram where the *x*-axis displays a sequence, while the *y*-axis shows the percentage of identity, in the 50–100% range, of gap-free segments of Blast Z-computed sequence alignments. The PIP presented in Figure 7 was created using PTPMaker [38] to compare the *S. solfataricus *genome sequence with itself. Unlike the dotplot, the PIP diagram does not sketch an overall view of the sequence: Figure 7 is a small snapshot of a 30-page document. Like dotplots, a PIP remains useful in locating repeated segments, but still conveys no information regarding the size and structural organization of these repeats: Figure 7 spans a CRISPR (region with high densities of conserved sequences), but it remains difficult to understand its structure.

**Figure 8:**
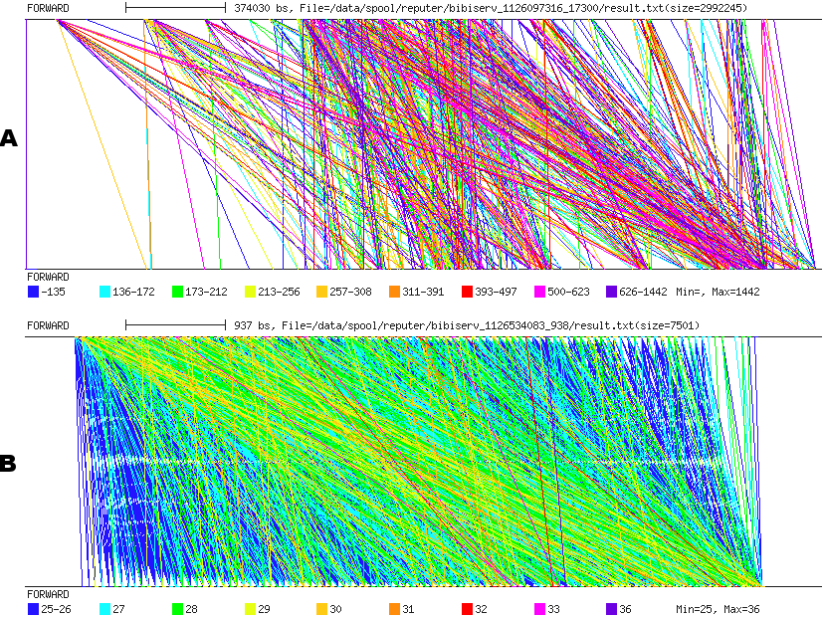
**Repeat graph**. A. Repeat graph of the 2.83 Mb sequence from *S. solfataricus*. B. Repeat graph of the *S. solfataricus *sequence region from coordinates 1,233,000 to 1,240,000, containing a CRISPR. Both repeat graphs are obtained by comparing the normal strand with itself.

Repeat graphs were produced using REPuter [30] (Figure 8). Like the dotplot and PIP, repeat graphs highlight the strong repetitive organization of the *S. solfataricus *genome sequence, but it remains quite difficult to depict the nature of this organization. For instance, when targeting CRISPR analysis, a repeat graph does not conveniently depict a structure of this type (Figure 8b). Unlike a *pygram*, at least two pictures must be produced when using a repeat graph: the first is used to compare the N strand with itself, while the second is used to compare the N strand with the RC strand.

## Conclusion

The pyramid diagram (or *pygram*) is a new visualization method that aims to summarize the complex hierarchical organization of repetitive sequence structures for either a single genomic sequence or across several sequences.

In contrast to similar existing tools, the *pygram *is not based on repeat pair display, and provides convenient graphical functions such as two-strand visualization, repeat frequency display, a zoom feature, repeat selection and annotation display. It therefore produces a better view of repeated sequences at all levels, from the complete genome sequence down to the nucleotide. Moreover, closely associating a viewer and a querying tool results in an efficient repeat browser, as illustrated in the examples on CRISPR investigation and DNA transfers in *Archaea *genomes.

The prototype developed uses a generalized suffix tree to produce eMRs. It achieves good linear performance (see *Methods*) with respect to the sequence size and the number of eMR occurrences to be handled, but the current application is limited to genome sequences containing no more than 50 million nucleotides on a computer with 4 Gb of RAM. During development, however, in the *pygram *browser implementation phase, the system that identifies the repeated sequences (in the present case, eMRs), was separated from the browser engine. This feature opens the *pygram *browsing infrastructure to other repeat models, in particular error-prone ones. In this way, *Pygram *could be used to perform the difficult job of analysing divergent sequences, a particularly crucial task in comparative genomics.

## Methods

### Implementation and performance of the *Pygram *application

**Figure 9:**
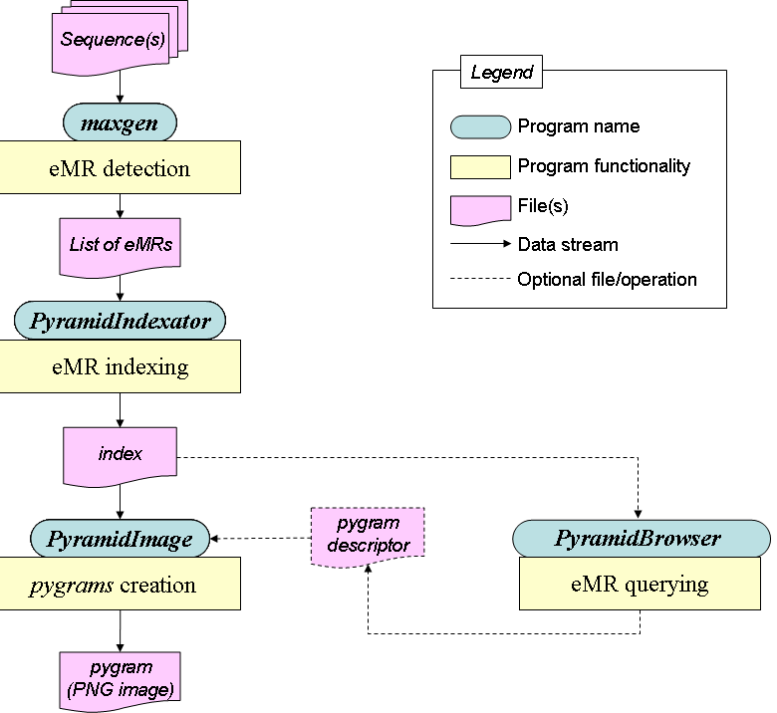
**Overall *Pygram *browser architecture**. Main components and data streams of the Pygram browser. See text for further details.

The *Pygram *environment consists of a suite of four complementary tools: *Maxgen, PyramidIndexator, PyramidImage *and *PyramidBrowser *(Figure 9). The software suite was benchmarked on a Dell Precision 370 computer running Red Hat Linux (Fedora Core 4). The computer features a Pentium IV, 3 GHz processor and has 4 Gb of RAM. GCC 3.2.2 was used to compile C source codes, and the J2SE 1.4.2-06 Software Development Kit from Sun Microsystems to compile and execute Java software.

*Maxgen *is an ANSI C software package that implements Gusfield's eMR detection algorithm on a generalized suffix tree (GST). This algorithm is capable of locating all eMRs in linear time and space, with respect to the sequence size, and presents the advantage of inserting the normal and reverse-complement sequences of each genome in a unique suffix tree. *Maxgen *proceeds in two steps. First, it analyses all real internal nodes of the GST, detecting all words that are eMRs. It then collects all occurrences of each eMR. The overall process runs at a rate of ~46 kbases/s, and the program uses an average of 17 bytes per sequence letter, which is slightly more than the highly space-efficient standard suffix tree application created by Kurtz [43]. Additional byte capacity is required to handle several sequences in a single suffix tree and detect eMRs. After running this software on a set of FASTA-format sequences, a text file is created containing all lexicographically ordered eMRs. Each line of this file represents a single eMR, along with all its positions in the analysed sequence(s).

*PyramidIndexator *is a Java program that converts the text file generated by *Maxgen *into two binary index files. The first index file stores an object representation of each eMR,, all eMRs being ordered lexicographically. The data file uses 36 bytes per eMR, (these bytes are used to store primary key and repeat size, type and colour), in addition to one byte per character to store the repeat sequence, and four bytes per occurrence to store the positions. The second index file stores an object representation of each eMR, for visualization purposes, all repeats being ordered by sequence position. The visualization file uses 17 bytes per repeat occurrence (to store primary key and repeat size and colour). Each of these binary files is associated with an index file to speed up data access. *PyramidIndexator *creates both index files in linear time and space with respect to the number of occurrences at a rate of ~310 k eMR, occurrences/s. Once the indexes have been created, *pygrams *can be created and index files queried on line. The current version of the index files requires a significant amount of memory to store each eMR,, since the data (primary key, repeat type, colour, position, etc.) are all encoded using the standard Java integer and colour classes. A future version of *PyramidIndexator *will optimize capacity requirements, an important issue for *Eukarya *genome visualization.

The colour scheme is computed using the sequence of each eMR: from each individual sequence, a hashcode is computed (using the standard Java API) which is in turn converted to RGB values.

*PyramidImage *is a Java application capable of creating *pygram *pictures. This program provides the visualization infrastructure necessary to explore repeated structures at various levels of magnification, from the highest level (i.e. the complete sequence) to the lowest level (a single nucleotide). This is achieved using a contextual zoom tool associated with a global viewer.

*PyramidImage *creates a *pygram *using the visualization index file generated by *PyramidIndexator: *the index file is scanned so that the larger repeats are displayed before the smaller ones. In this way, the larger pyramids do not hide the smaller ones. To display each repeat, pyramids are produced in increasing sequence position order. *PyramidImage *runs in linear time and space at a rate of ~165 k eMR occurrences/s.

*PyramidImage *receives input from two files. The first is the visualization binary file created by *PyramidIndexator*. The second is an optional text file, referred to as a *pygram *descriptor, that contains drawing and filtering parameters. If no *pygram *descriptor is provided, *PyramidImage *creates a *pygram *for the entire genome sequence, displaying all eMRs reported in the index file. On the other hand, if a descriptor is provided, these parameters can be controlled to produce *pygrams *with various layouts (see examples in Figure 2). The drawing parameters include:

• image size,

• region of the sequence to display,

• standard or logarithmic *pygram*,

• number of lenses to produce,

• eMR to highlight.

For each lens, the descriptor can be used to specify the location of the lens within the sequence, *x- *and *y*-axis magnifying factors, the sequence coordinate ruler, and whether or not to display sequence letters. The filtering parameters for eMRs include:

• size range,

• occurrence range,

• sequence location.

*PyramidImage *can also display annotations on the *pygram*. This feature can be used to display either known genome annotations or user-defined ones. Figure 2D presents a full-featured example of *pygram *visualization functionality: two different regions of the same sequence are presented side-by-side, along with a zoom lens, several features underlying a genomic structure of interest and a selected eMR. The DNA sequence is also displayed inside the lens, and a *y*-axis magnifying factor is applied to achieve better structure magnification.

*PyramidBrowser *is Java software designed to query the binary files created by *PyramidIndexator*. This tool can be used to select specific eMRs according to their size, number of occurrences and location on the sequence. The information from *PyramidBrowser *can be entered as filtering parameters in the *pygram *descriptor.

### Size of the eMR occurrence index file

Creating an index file for all observed instances of an eMR in a genome sequence can be difficult to compute, since the number of occurrences (number of locations) is not linear with respect to the number of eMR types (number of different words) observed in a sequence. For instance, the sequence *CA*^*n*^*GA*^*n*^*T *contains exactly n maximal repeats (different words), namely *A*^*k *^(*k *= 1, *n*), and the number of occurrences of all these repeats is ∑i=1n2i=n(n+1)
 MathType@MTEF@5@5@+=feaafiart1ev1aaatCvAUfKttLearuWrP9MDH5MBPbIqV92AaeXatLxBI9gBaebbnrfifHhDYfgasaacH8akY=wiFfYdH8Gipec8Eeeu0xXdbba9frFj0=OqFfea0dXdd9vqai=hGuQ8kuc9pgc9s8qqaq=dirpe0xb9q8qiLsFr0=vr0=vr0dc8meaabaqaciaacaGaaeqabaqabeGadaaakeaadaaeWaqaaiabikdaYiabdMgaPjabg2da9iabd6gaUjabcIcaOiabd6gaUjabgUcaRiabigdaXiabcMcaPaWcbaGaemyAaKMaeyypa0JaeGymaedabaGaemOBa4ganiabggHiLdaaaa@3D05@.

Since the task covers millions of different words, quadratic behaviour such as this is computationally intractable. Experiments were therefore conducted on several genomes to study the practical impact of the relationship between the number of eMR occurrences and the number of eMR types. Two scenarios were tested empirically: a linear relationship and a quadratic relationship. The ratio for each case was computed as:

• the ratio between eMR occurrences and the number of eMR types (a) and,

• the ratio between eMR occurrences
 MathType@MTEF@5@5@+=feaafiart1ev1aaatCvAUfKttLearuWrP9MDH5MBPbIqV92AaeXatLxBI9gBaebbnrfifHhDYfgasaacH8akY=wiFfYdH8Gipec8Eeeu0xXdbba9frFj0=OqFfea0dXdd9vqai=hGuQ8kuc9pgc9s8qqaq=dirpe0xb9q8qiLsFr0=vr0=vr0dc8meaabaqaciaacaGaaeqabaqabeGadaaakeaadaGcaaqaaiabdwgaLjabd2eanjabdkfasjabbccaGiabd+gaVjabdogaJjabdogaJjabdwha1jabdkhaYjabdkhaYjabdwgaLjabd6gaUjabdogaJjabdwgaLjabdohaZbWcbeaaaaa@404C@ and the number of eMR types (b).

**Figure 10:**
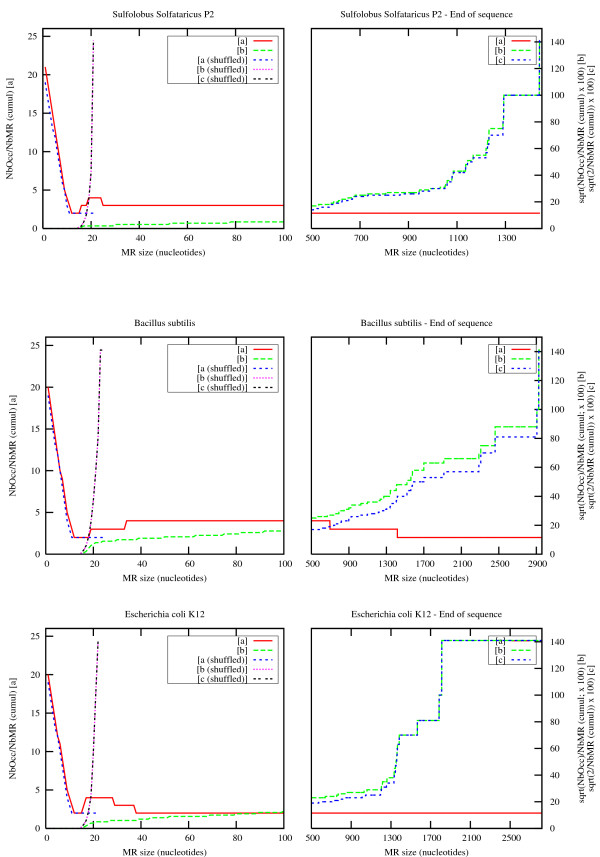
**Number of observed eMRs and their occurrences in *Sulfolobus solfataricus, Bacillus subtilis *and *Escherichia coli***. On each plot, red and green curves refer to eMRs located in the original genome sequence, while blue and purple curves refer to eMRs located in shuffled sequences computed using shuffleseq from the EMBOSS package [42]. For each curve, *NbMR *refers to the total number of eMRs observed for a given size and above (cumulative values), and *NbOcc *refers to the grand total of eMR occurrences observed for a given size and above (cumulative values). [a] displays ∑NbOcc∑NbMR
 MathType@MTEF@5@5@+=feaafiart1ev1aaatCvAUfKttLearuWrP9MDH5MBPbIqV92AaeXatLxBI9gBaebbnrfifHhDYfgasaacH8akY=wiFfYdH8Gipec8Eeeu0xXdbba9frFj0=OqFfea0dXdd9vqai=hGuQ8kuc9pgc9s8qqaq=dirpe0xb9q8qiLsFr0=vr0=vr0dc8meaabaqaciaacaGaaeqabaqabeGadaaakeaadaWcaaqaamaaqaeabaGaemOta4KaemOyaiMaem4ta8Kaem4yamMaem4yamgaleqabeqdcqGHris5aaGcbaWaaabqaeaacqWGobGtcqWGIbGycqWGnbqtcqWGsbGuaSqabeqaniabggHiLdaaaaaa@3BE7@, [b] displays 100. ∑NbOcc∑NbMR
 MathType@MTEF@5@5@+=feaafiart1ev1aaatCvAUfKttLearuWrP9MDH5MBPbIqV92AaeXatLxBI9gBaebbnrfifHhDYfgasaacH8akY=wiFfYdH8Gipec8Eeeu0xXdbba9frFj0=OqFfea0dXdd9vqai=hGuQ8kuc9pgc9s8qqaq=dirpe0xb9q8qiLsFr0=vr0=vr0dc8meaabaqaciaacaGaaeqabaqabeGadaaakeaadaWcaaqaamaakaaabaWaaabqaeaacqWGobGtcqWGIbGycqWGpbWtcqWGJbWycqWGJbWyaSqabeqaniabggHiLdaaleqaaaGcbaWaaabqaeaacqWGobGtcqWGIbGycqWGnbqtcqWGsbGuaSqabeqaniabggHiLdaaaaaa@3C02@, and [c] displays 2∑NbMR
 MathType@MTEF@5@5@+=feaafiart1ev1aaatCvAUfKttLearuWrP9MDH5MBPbIqV92AaeXatLxBI9gBaebbnrfifHhDYfgasaacH8akY=wiFfYdH8Gipec8Eeeu0xXdbba9frFj0=OqFfea0dXdd9vqai=hGuQ8kuc9pgc9s8qqaq=dirpe0xb9q8qiLsFr0=vr0=vr0dc8meaabaqaciaacaGaaeqabaqabeGadaaakeaadaWcaaqaamaakaaabaGaeGOmaidaleqaaaGcbaWaaabqaeaacqWGobGtcqWGIbGycqWGnbqtcqWGsbGuaSqabeqaniabggHiLdaaaaaa@34A9@.

These values were compared for *Archaea *and *Bacteria *genomes and for random sequences extracted from shuffled versions of these genomes. Figure 10 displays an overview of the results for three genomes (one thermophilic *Archaea*, one *Gram*^+ ^*Bacteria *and one *Gram*^- ^*Bacteria*). It presents cumulative curves for eMR of increasing size. In other words, for each threshold *x *representing the minimum size of the observed maximal repeats, the curves display the ratios for all repeats whose size is greater than or equal to *x*.

The ratio trends are remarkably similar in all cases.

First of all, the left side of the [a] curves and [b] curves is the same for the normal and shuffled versions of the genomes. This tends to show that short maximal repeats occur at a frequency that depends only on the sequence structure. In contrast, long maximal repeats occur more often in normal genomes than in their shuffled counterpart. Furthermore, they continue to occur at an almost constant rate for quite large word sizes, whereas the maximum size of maximal repeats remains less than 25 nucleotides in random sequences of the same length. Note that the final behaviour of [b] curves simply follows sqrt(2/NbMR) since Nbocc is proportional to NbMR: [c] curves clearly show this fact. The last value is sqrt(2), corresponding to one MR with two occurrences.

The second important observation is that the overall trend for the number of occurrences is quadratic, as expected for very short words (the left side of the [b] curves is flat), then decreases rapidly and becomes almost linear for long words (the right side of the [a] curves is almost flat). The number of occurrences of significant maximal repeats (those that can be distinguished from randomly occurring ones) therefore remains comparable with the number of maximal repeat types, which means that a systematic analysis of these eMRs may reasonably be attempted along a genome.

## Availability and Requirements

Pygram tools (precompiled binaries, documented source code and user manuals) are distributed under the CeCILL (CRA-CNRS-INRIA Logiciel Libre) free software license and are available at .

## Authors' contributions

PD designed the *pygram *visualization method. ASV and PD wrote the software source code. FM performed the sequence analysis. PD and JN wrote the manuscript. All authors read and approved the final manuscript.
